# Angiopoietin/Tie2 Dysbalance Is Associated with Acute Kidney Injury after Cardiac Surgery Assisted by Cardiopulmonary Bypass

**DOI:** 10.1371/journal.pone.0136205

**Published:** 2015-08-26

**Authors:** Rianne M. Jongman, Jan van Klarenbosch, Grietje Molema, Jan G. Zijlstra, Adrianus J. de Vries, Matijs van Meurs

**Affiliations:** 1 Department of Anesthesiology, University Medical Center Groningen, Groningen, the Netherlands; 2 Department of Pathology and Medical Biology, Medical Biology Section, University Medical Center Groningen, Groningen, the Netherlands; 3 Department of Critical Care, University Medical Center Groningen, Groningen, the Netherlands; 4 Department of Anesthesiology, University Medical Center Utrecht, Utrecht, the Netherlands; UCL Institute of Child Health, UNITED KINGDOM

## Abstract

**Introduction:**

The pathophysiology of acute kidney injury (AKI) after cardiac surgery is not completely understood. Recent evidence suggests a pivotal role for the endothelium in AKI. In experimental models of AKI, the endothelial specific receptor Tie2 with its ligands Angiopoietin (Ang) 1 and Ang2 are deranged. This study investigates their status after cardiac surgery, and a possible relation between angiopoietins and AKI.

**Methods:**

From a cohort of 541 patients that underwent cardiac surgery, blood and urine was collected at 5 predefined time points. From this cohort we identified 21 patients who had at least 50% post-operative serum creatinine increase (AKI). We constructed a control group (n = 21) using propensity matching. Systemic levels of Ang1, Ang2, and sTie2 were measured in plasma and the AKI markers albumin, kidney injury molecule-1 (KIM-1) and N-acetyl-beta-D-glucosaminidase (NAG) were measured in the urine.

**Results:**

Ang2 plasma levels increased over time in AKI (from 4.2 to 11.6 ng/ml) and control patients (from 3.0 to 6.7 ng/ml). Ang2 levels increased 1.7-fold more in patients who developed AKI after cardiac surgery compared to matched control patients. Plasma levels of sTie2 decreased 1.6-fold and Ang1 decreased 3-fold over time in both groups, but were not different between AKI and controls (Ang1 *P* = 0.583 and sTie2 *P* = 0.679). Moreover, we found a positive correlation between plasma levels of Ang2 and urinary levels of NAG.

**Conclusions:**

The endothelial Ang/Tie2 system is in dysbalance in patients that develop AKI after cardiac surgery compared to matched control patients.

## Introduction

Acute kidney injury (AKI) is a major complication after cardiac surgery and is correlated with an increase in post-operative mortality [1]. The incidence of AKI after cardiac surgery varies ranging from 14–50% depending on the definition used [2]. Renal replacement therapy is necessary in about 3% of the patients with post-operative AKI [2]. Even if kidney function normalizes after the initial hospital stay, AKI is still correlated with long-term mortality and morbidity[3]. Understanding the pathophysiological mechanism of AKI is necessary to intervene in the development of AKI.

There is increasing evidence that the renal endothelium plays a crucial role in AKI development, by expressing endothelial adhesion molecules leading to leukocyte recruitment and inflammation, and loss of microvascular integrity leading to vascular leakage [4]. The Angiopoietin/Tie2 receptor system is a regulator of microvascular inflammation and vascular leakage and its importance in critical illness has previously been reviewed [5]. Angiopoietin-1 (Ang1) and Angiopoietin-2 (Ang2) are signaling molecules that bind to their specific endothelial tyrosine kinase receptor Tie2, which is constitutively expressed in the adult vasculature [6]. The Tie2 agonist Ang1 is produced by pericytes. In a quiescent state of the endothelium, binding of Ang1 to the Tie2 receptor leads to Tie2 phosphorylation which maintains endothelial cell integrity. The Tie2 antagonist Ang2 is a competitive antagonist of Ang1 produced by endothelial cells and is stored in Weibel-Palade bodies [7]. Upon stress and pro inflammatory stimulation, Ang2 is released from endothelial cells into the circulation [8], where it binds to its receptor and reduces vascular integrity. Although the angiopoietins have long been considered to be the dynamic factors of the system, recent evidence suggests a reduction of Tie2 receptor expression levels on endothelial cells in animal models of critical illness [9]. Tie2 can be shed from the membrane, and soluble Tie2 (sTie2) and can act as a scavenger receptor for the angiopoietins.

It has been shown that the Ang/Tie2 system is deranged in septic patients [10,11] and after cardiac surgery [12,13]. Increased plasma levels of Ang2 have a prognostic value for mortality and morbidity in patients with chronic kidney disease [14,15]. In mice that developed AKI after sepsis, Ang2 and Tie2 messenger RNA levels in the kidney changed [16]. Therefore, we hypothesized that the Ang/Tie2 system is associated with the development of post-operative AKI reflected by changes in systemic plasma levels of Ang1, Ang2 and sTie2.

## Materials and Methods

### Patients

This observational study consisted of 541 patients, a subgroup of a larger randomized prospective multicenter clinical trial that investigated the effects of intraoperative cell salvage and leukocyte depletion on allogeneic blood exposure during cardiac surgery. The trial was registered at http://www.trialregister.nl/trialreg/admin/rctview.asp?TC=244, under number ISRCTN58333401 and was approved by the Medical Ethical Committee of the University Medical Center Groningen, The Netherlands. Written informed consent was obtained from all patients included in the study. The main results of this study were previously published [17].

We identified 21 patients (4% of the total study population) who had a serum creatinine increase of at least 50% in the immediate post-operative period. According to the RIFLE criteria, this indicates AKI [18]. Urine output to assess AKI was not used in this study, as these data were not available for the whole patient group. Propensity score analysis, based on a forward stepwise logistic regression model was used to match the AKI patients in a one to one ratio with 21 patients that did not meet this criteria for AKI. As covariates, the baseline demographic variables were used, and in particular preoperative serum creatinine, preoperative hemoglobin level and estimated renal function as assessed by the Cockcroft-Gault formula. Procedure related variables were also entered into the model. These matched control pairs were created using a macro for SPSS (Version 18.0, IBM Chicago, Ill.), which compared the nearest propensity scores of the patients without AKI with the scores of the patients with AKI. The standardized difference between the groups were calculated to assess the imbalance of covariates (Table 1).

**Table 1:** Patient demographics.

	Overall (n = 541)	standardized differences overall vs AKI	AKI (n = 21)	standardized differences (AKI vs control)	Control (n = 21)	*p*-value (AKI vs control)
**Age (years)**	66 ± 9.6	0.52	71 ± 8.4	0.47	68 ± 8.7	0.15
**Height (cm)**	173 ± 9	-0.35	170 ± 10	0.10	169 ± 10	0.87
**Weight (kg)**	82 ± 13	0.75	83 ± 14	0.21	80 ± 14	0.46
**Male (n (%))**	400 (74)	-0.13	14 (67)	0.25	11 (52)	0.53
**EuroSCORE**	4.4 ± 3.1	0.94	7.3 ± 4.3	0.44	5.7 ± 3.3	0.18
***Coexisting illness (%)***						
**Myocardial infarction**	24	0.00	24	0.10	20	1.00
**PCI**	12	0.15	19	0.18	15	1.00
**Hypertension**	48	0.02	48	-0.15	60	0.76
**Stroke**	5	0.12	9	0.13	5	1.00
**Atrial fibrillation**	12	0.34	28	0.30	14	0.45
**Diabetes**	24	0.18	33	0.17	25	0.73
**COPD**	13	0.05	14	0.00	15	1.00
***Medication (%)***						
**Aspirin <3 day**	51	-0.29	52	0.08	48	0.95
**Beta blocker**	69	-0.03	67	0.00	67	1.00
**Calcium-antagonist**	28	0.17	38	0.00	38	1.00
**ACE inhibitor**	40	0.46	67	0.59	33	0.06
***Preoperative data***						
**Hemoglobin (mmol/L)**	7.6 ± 0.9	-0.67	7.0 ± 0.9	0.00	7.0 ± 0.7	0.94
**Platelets (x10** ^**9**^ **/L)**	205 ± 57	0.09	210 ± 101	0.13	220 ± 54	0.72
**Creatinine (mmol/L)**	87 ± 27	0.11	90 ± 28	0.00	90 ± 26	0.98
**Cockroft (mL/min)**	89 ± 28	-0.38	79 ± 26	0.00	78 ± 26	0.96
***Procedures***						
**CABG (n (%))**	338 (62)		10 (48)		13 (62)	
**Valve (n (%))**	134 (25)		5 (24)		6 (29)	
**CABG + valve (n (%))**	69 (13)		6 (29)		2 (9)	
***CPB management***						
**Aortic cross-clamp (min)**	66 ± 28	0.50	80 ± 30	0.24	73 ± 28	0.44
**CPB time (min)**	103 ± 42	0.45	122 ± 42	0.06	119 ± 53	0.83
**Hemoglobin CPB (mmol/L)**	4.9 ± 0.74	-0.68	4.4 ± 0.60	-0.16	4.5 ± 0.63	0.63

PCI: percutaneous coronary intervention, COPD: chronic obstructive pulmonary disease, CABG: coronary artery bypass grafting, CPB: cardiopulmonary bypass. Data are presented as mean ± SD or as a percentage where indicated. Standardized differences are shown between the whole patient group (overall) and the patients with acute kidney injury (AKI), and between AKI and a matched control group (control). *P*-values are given for AKI versus control group.

### Procedures

Patients underwent coronary artery bypass grafting, valve replacement or combined procedures with use of cardiopulmonary bypass (CPB). The CPB circuit was primed with 1000 mL lactated Ringer’s solution and 500 mL hydroxyethylstarch 10% (Fresenius, Bad Homburg, Germany). All patients received standard anesthesia.

### Blood sampling

Blood and urinary samples were obtained from each patient at 5 predefined moments: after induction of anesthesia, at the end of the surgery, after 3h in the intensive care unit, on the morning of the first post-operative (day 1) and at the morning of the second (day 2) post-operative day. Blood samples were centrifuged at 1000g for 10 minutes, and the plasma was stored in small portions at -80°C for analysis.

### Measurements of plasma levels of soluble factors of the Ang/Tie2 system

Plasma levels of Ang1, Ang2 and sTie2 were measured with Quantikine ELISA kits (R&D Systems, Minneapolis, MN, USA), according to the manufacturer’s instructions.

### Measurements of markers of renal injury

Serum creatinine was measured with an enzymatic creatinine assay (Roche Diagnostics GmbH, Mannheim, Germany). The urinary levels of Kidney Injury Molecule-1 (KIM-1) were measured with Quantikine ELISA kits (R&D Systems) according to the manufacturer’s instructions. Albumin levels in urine were determined by the bromcresol green method[19] and N-acetyl-β-D-glucosaminidase (NAG) levels were measured by means of a substrate assay [20].

### Statistical analysis

Continuous data were analyzed using Student’s t-test or the Mann-Whitney U-test where appropriate. Categorical variables were analyzed using Fisher exact test. Two-way ANOVA with repeated measurements was used to compare serial data. Correlations between variables were analyzed by the Pearson correlation coefficient. All statistical analyses were performed using SPSS (Version 18.0, IBM Chicago, Ill). A *P*-value < 0.05 was considered significant.

## Results

As per selection criterion, 21 patients with AKI had higher serum creatinine levels than the propensity-matched controls. Eleven patients had Risk of renal failure (mean post-operative serum creatinine level 149 ± 43 μmol/L), 9 patients had renal Injury (204 ± 54μmol/L) and 1 patient was classified as Failure (265 μmol/L). The pre- and intraoperative patient demographics in the total patient cohort, the AKI group and the matched control group are presented in Table 1 and in Table 2, the transfusion and post-operative data are presented. The EuroSCORE in the AKI group was 7.3% vs 5.7% in the control group, while in the overall cohort the EuroSCORE was 4.4%, indicating that patients with AKI had a higher preoperative procedure risk than the total patient population. It is of note that, although not statistically different (*P* = 0.06), 67% of the AKI patients were on ACE inhibitors whereas this was the case in 33% of the patients in the control group. The standardized differences between AKI patients and the matched control group indicate a reasonable match for AKI risk factors.

**Table 2:** Transfusion and post-operative data.

	Overall (n = 541)	AKI (n = 21)	Control (n = 21)	*p*-value (AKI vs control)
**Patients received RBC first 24 hrs (n (%))**	223 (41)	16 (76)	12 (57)	0.32
**Patients received FFP (n (%))**	89 (16)	6 (29)	7 (33)	1.00
**Patients received platelets (n (%))**	80 (15)	5 (24)	6 (29)	1.00
**Chest tube loss first 12 hrs (mL)**	682 ± 570	920 ± 696	1087 ± 1611	0.67
**Hemoglobin day 1 (mmol/L)**	6.4 ± 0.84	6.1 ± 0.43	6.1 ± 0.59	0.91
**Colloid (mL)**	1083 ± 805	1569 ± 840	1467 ± 868	0.73
**LOS ICU (days)**	1.7 ± 3.7	6.3 ± 12.5	1.5 ± 1.1	0.10
**LOS hospital (days)**	11.2 ± 9.4	21 ± 19.5	12 ± 9.4	0.05
**Reexploration (n (%))**	32 (6)	3 (14)	3 (14)	1.00
**Myocardial infarction (n (%))**	24 (4)	1 (5)	1 (5)	1.00
**Stroke (n (%))**	9 (2)	0 (0)	0 (0)	1.00
**Atrial fibrillation (n (%))**	168 (31)	10 (48)	8 (38)	0.54
**Infection (n (%))**	73 (13)	7 (33)	2 (10)	0.06

RBC: red blood cell concentrate, FFP: fresh frozen plasma, LOS: length of stay in the ICU. Data are presented as mean ± SD or as a percentage for the whole patient group (overall), the patients with acute kidney injury (AKI), and a matched control group (control). Length of stay is expressed as median and interquartile range. *P*-values are given for AKI versus control group.

**Fig 1:**
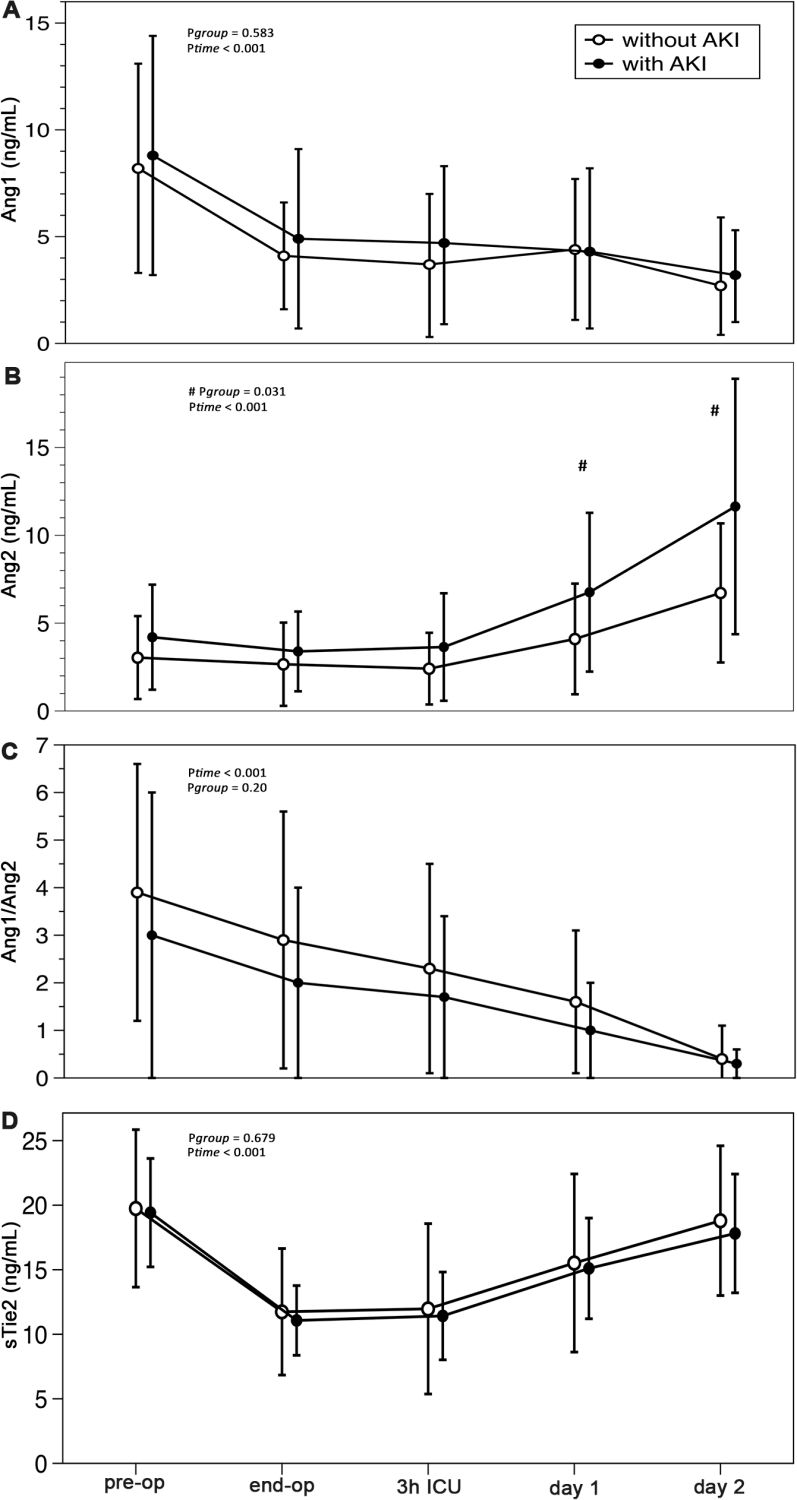
Plasma levels of Angiopoietin-1, Angiopoietin-2 and soluble Tie2 in patients with and without acute kidney injury after cardiac surgery. **A:** Angiopoietin-1 (Ang1); **B:** Angiopoietin-2 (Ang2); **C:** Ang1/Ang2; **D:** soluble Tie2 (sTie2). AKI: **acute kidney injury. Time points are before (pre-op) and at the end of the operation (end-op), after 3 hours in the intensive care unit (3h ICU) and on the first and second post-operative day (day 1 and day 2).** P-values are given for differences between the groups (^#^ P_*group*_) and differences in time (P_*time*_) using two-way ANOVA with repeated measurements.

The plasma levels of Ang1, Ang2, and sTie2 changed over time in AKI and in control patients (Fig 1). Ang1 levels decreased over time in both groups from 8.5 ng/mL to 2.6 ng/mL (3-fold) (*P*<0.01). This decrease occurred at the end of the operation in both groups and did not return to baseline levels within 48h after surgery. Ang2 increased over time in both groups (control: from 3.0 ± 2.4 ng/mL tot 6.7 ± 2.4 ng/ml; AKI: from 4.2 ± 3.0 ng/mL to 11.6 ± 7.6 ng/ml) (*P* = 0.013). This increase was 1.6-fold higher in patients with AKI at day 1 and 1.7-fold higher at day 2 after surgery. The plasma levels of Ang2 did not correlate with CPB time. Plasma levels of sTie2 showed a drop of 1.6-fold at the end of the operation (from 19.5 to 11.3 ng/ml) and returned to baseline levels (18.3 ng/ml) within 48h after surgery. No interaction was found for both Ang1 (*P* = 0.583) and sTie2 (*P* = 0.679) between AKI patients and control patients.

**Fig 2:**
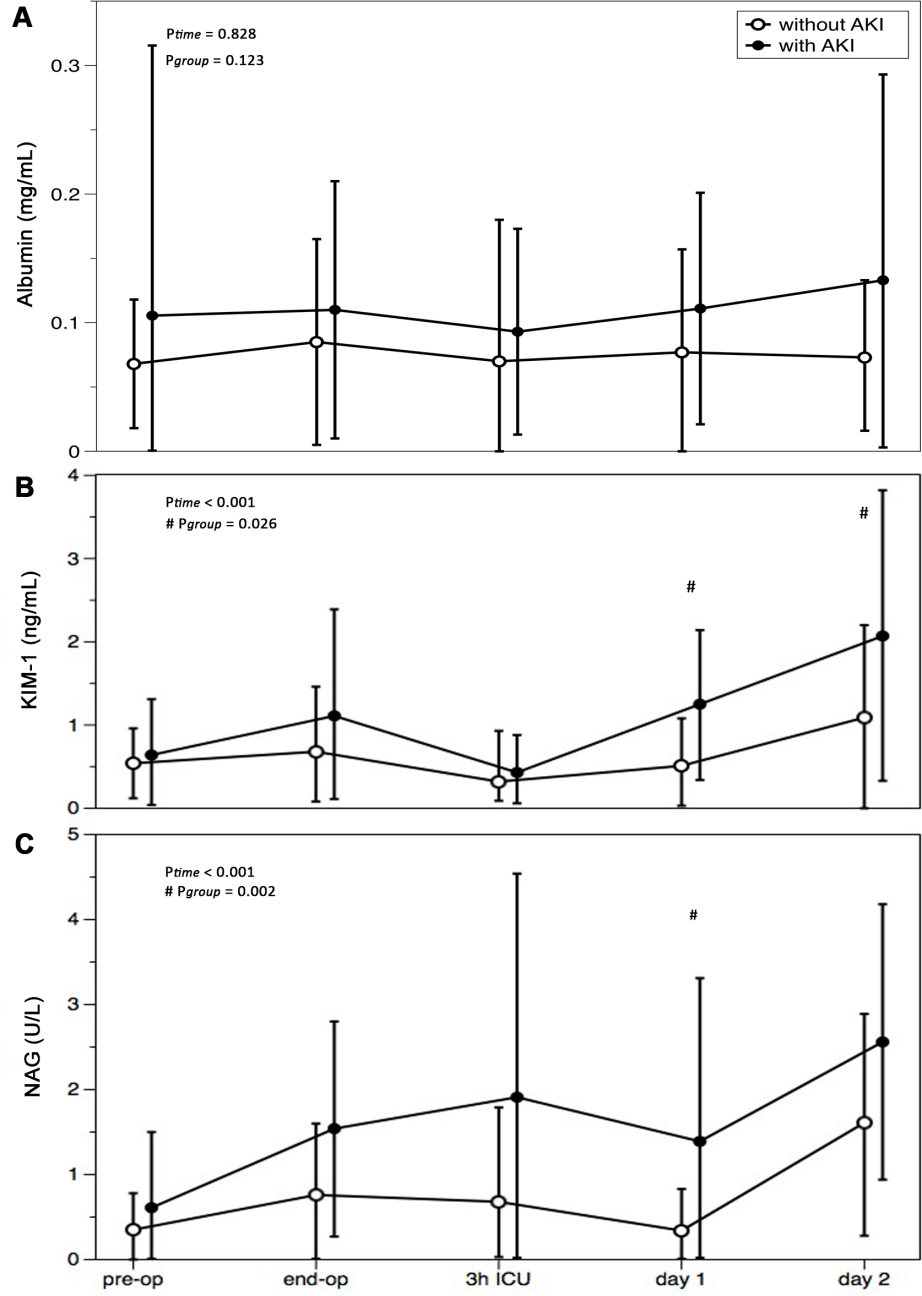
Urinary levels of markers for renal injury in patients with and without acute kidney injury after cardiac surgery. **A:** Albumin; **B:** Kidney Injury Molecule-1 (KIM-1); **C:** N-acetyl-β-D-glucosaminidase (NAG). AKI: acu**te kidney injury. Time points are before (pre-op) and at the end of the operation (end-op), after 3 hours in the intensive care unit (3h ICU) and on the first and second post-operative day (day 1 and day 2).** P-values are given for differences between the groups (^#^ P_*group*_) and differences in time (P_*time*_). using two-way ANOVA with repeated measurements.

The time course of the urinary markers albumin, KIM-1 and NAG is shown in Fig 2. Albumin levels did not change over time and we did not find an interaction between the groups. KIM-1 levels increased 3.2-fold (from 0.64 ± 0.67 ng/ml to 1.84 ± 1.75 ng/ml) in the AKI group, whereas in the control group the KIM-1 levels increased 2-fold (from 0.54 ± 0.42 ng/ml to 1.09 ± 1.14 ng/ml). KIM-1 levels were 2.4-fold higher in the AKI group compared to controls at day 1 and 1.9-fold higher at day 2. There was a difference between the groups over time (*P* = 0.05). NAG levels increased 4.2-fold (from 0.61 ± 0.88 U/l to 2.56 ± 1.62 U/l) in the AKI group, whereas in the control group the NAG levels increased 4.5-fold (from 0.35 ± 0.43 U/l to 1.54± 1.33 U/l). NAG levels were 4.1-fold higher in the AKI group compared to control at day 1 (*P* = 0.001).

**Fig 3:**
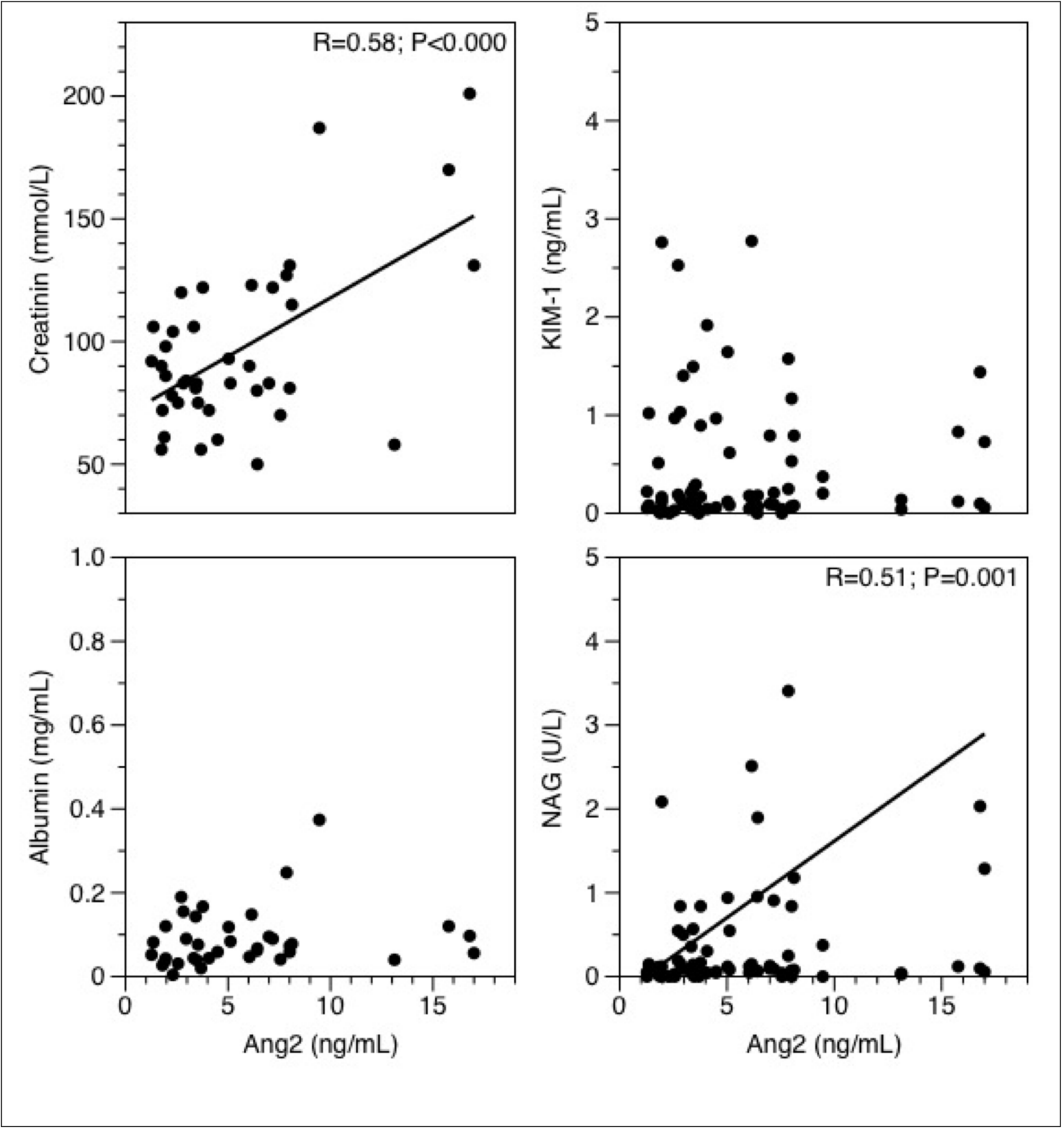
Correlations between plasma Ang2 levels and urinary levels of markers renal injury on the first post-operative day in patients after cardiac surgery. **A:** serum creatinine; **B: urine** Kidney Injury Molecule-1 (KIM-1); **C:** urine Albumin; **D:** urine N-acetyl-β-D-glucosaminidase (NAG); Correlation (R) is given when significant. Statistics are analyzed using the Pearson correlation coefficient.

Because the plasma levels of Ang2 of the AKI and control patients showed a different post-operative time course, we next studied whether correlations were present between Ang2 plasma levels and markers of AKI (Fig 3). We found a positive correlation between the plasma levels of Ang2 and serum creatinine on the first post-operative day. We furthermore observed a positive correlation between the plasma levels of Ang2 and urinary NAG levels, while no correlation was observed between the plasma levels of Ang2 and urinary KIM-1 levels and between the plasma levels of Ang2 and urinary albumin concentrations.

## Discussion

In this study we focused on changes in plasma levels of the Ang/Tie2 system in cardiac surgical patients who developed post-operative AKI. We demonstrated that higher systemic plasma levels of Ang2 were present in patients who develop AKI after cardiac surgery compared to matched controls. We found decreased systemic levels of Ang1 and sTie2 in both groups at the end of surgery, independent of the development of AKI. Plasma levels of sTie2 returned to baseline levels two days after surgery, whereas levels of Ang1 did not. Ang2 plasma levels correlated with the urinary AKI marker NAG.

Increased systemic levels of Ang2 have been described in patients after cardiac surgery [12,13] and have been associated with the duration of CPB [21]. We did not find such an association. We matched for aortic cross-clamp time and thus reduced outliers, which may affect the correlations that were found in two small scale studies [13,21]. The higher plasma levels of Ang2 in our patients with AKI may be explained by release of Ang2 from the Weibel-Palade bodies from the activated endothelium, rather than by a diminished excretion of Ang2. Although an inverse correlation between plasma Ang2 and glomerular filtration rate has been described [22,23]. It should also be noted that in insulin-dependent patients with diabetes type 2 and albuminuria, urine Ang2 levels increase while urine Ang1 levels decrease [24] suggesting that the higher levels of Ang2 in our AKI patients cannot be completely explained by a diminished excretion of Ang2. We did not find a correlation between the plasma levels of Ang2 and albuminuria. This has been found in chronic kidney disease [22], but may not be the case in the acute setting of our study. Plasma levels of Ang2 correlated with urinary NAG levels in our patients, which suggests a relation between systemic levels of Ang2 and AKI, but we did however not find a correlation with urinary KIM levels that could support such a relation.

Endothelial behavior in cardiac patients is controlled by multiple cytokines [25] vascular growths factors like the VEGF/VEGFR system and Ang/Tie2 system [12], hypoxia [26] and local blood flow [27]. However, in our previous study we found low levels in VEGF (about 50 ng/mL). It is therefore likely that Ang2 is mainly associated with apoptosis and vessel disintegration [7].

In healthy conditions, Ang1 binds to the Tie2 receptor, resulting in phosphorylation of the Tie2 receptor leading to reduced endothelial cell activation and reduced vascular leakage[5]. Ang2 is a competitive antagonist of Ang1. During inflammatory conditions, the pro inflammatory cytokine TNF-α, induces Ang2 release from endothelial cells [28]. Ang2 is then rapidly released from the Weibel-Palade bodies [29]. Ang2 sensitizes the endothelial cells to inflammatory mediators [30]. Ang2 acts as an antagonist of Ang1 and binds to the Tie2 receptor with higher affinity than Ang1, leading to dephosphorylation of the Tie2 receptor. In our study, we found reduced levels of Ang1 and increased levels of Ang2. Given that endothelial activation occurs within 30min after the induction of hemorrhagic shock [31], that Ang2 is rapidly released from endothelial cells, and that kidney injury cannot be measured at that time yet, it is likely that AKI is a consequence of endothelial dysfunction that could lead to vascular leakage.

In kidney biopsies of patients who died with AKI in the ICU mRNA levels of Ang1, Ang1/Ang2 ratio, and Tie2 were decreased immediate post-mortem [10] It is tempting to speculate that these changes also take place in kidneys of CPB patients who develop AKI. However, the functional consequences of this Ang1/Ang2 dysbalance on vascular inflammation and leakage in the kidney cannot be examined using soluble levels of angiopoietins and Tie2 in cardiac surgical patients. Based on the fast response of endothelial cells to release Ang2 in the blood in healthy volunteers injected with LPS [8], we expected an increase in Ang2 plasma levels at an early time point after cardiac surgery, but we found increased Ang2 plasma levels on the first post-operative day, and the Ang2 levels gradually increased more on the second post-operative day. Currently we do not know which organs contribute most to the increased Ang2 levels. It is likely that organs with a high vascular density play a crucial role. Our study suggests that destabilization of the Ang/Tie2 system in mature, non-remodeling vessels might be a new target to prevent AKI after cardiac surgery. Different pharmacological strategies to intervene in the Ang/Tie2 system have already been used in the past in animal models [32–34]. It would be worthwhile to use a rodent CPB model to compare circulatory markers with tissue markers and study local effects on Tie2 phosphorylation, endothelial inflammation, vascular leakage in the kidney and kidney function. Novel drug therapies that influence the Ang/Tie2 can be tested in these animal models. Taken together, influencing the Ang/Tie2 signaling pathway might be a therapeutic target for the prevention or treatment of AKI post CPB.

Our study has several limitations. First, from our overall cohort of 541 patients, we found only 21 patients (4%) who developed AKI. We selected the patients only on elevations of serum creatinine according to the RIFLE criteria, and did not use urine output criteria. These data were not available for the whole patient cohort as at the time of inclusion of the patients, electronic data management systems were not universally used. If we had applied the elevations of serum creatinine according to the AKIN criteria (that also take into account an absolute creatinine increase more than 26,2 μmol/l), an additional 29 patients, all in AKIN stage 1, would have met the criteria of AKI (9,3%). Using creatinine levels only, AKI incidence is underestimated [35]. Second, we cannot compare our patients with other patients in the ICU, because we have no complete dataset to assess APACHE or SOFA score. Third, we did not analyze the whole patient cohort, therefore propensity matching was necessary to find patients that could serve as non-AKI controls. Fourth, the absolute levels of urine albumin, KIM-1 and NAG were low, most likely because of the development of the relatively mild AKI. However, degradation of proteins in urine stored for longer periods cannot be excluded [36]. And finally, patients in the AKI group were well matched with the control group, except for the use of ACE inhibitors.

## Conclusions

Cardiac surgery with the use of CPB leads to systemic changes in molecules of the Ang/Tie2 system, and these changes are more pronounced in patients that develop AKI. Ang2 levels increased more in patients who developed AKI after cardiac surgery, compared to matched controls and correlated with urine NAG levels. Animal studies aimed at restoring the Ang1/Ang2 balance as a tool to prevent AKI are warranted. These studies will extend our knowledge on therapies for patients with AKI after cardiac surgery.
